# The Perceived Value of Behavioural Traits in Australian Livestock Herding Dogs Varies with the Operational Context

**DOI:** 10.3390/ani9070448

**Published:** 2019-07-16

**Authors:** Jonathan Early, Elizabeth Arnott, Bethany Wilson, Claire Wade, Paul McGreevy

**Affiliations:** 1 Sydney School of Veterinary Science, Faculty of Science, University of Sydney, Sydney, NSW 2006, Australia; 2 Sydney School of Life and Environmental Sciences, Faculty of Science, University of Sydney, Sydney, NSW 2006, Australia

**Keywords:** herding, livestock, working dog, survey, traits, boldness

## Abstract

**Simple Summary:**

Information on Australian livestock herding dogs and their handlers and breeders is limited. This study aimed to collate baseline information on how handlers and breeders value various behavioural traits relevant to the work of these dogs. A survey was presented to explore herding dog behaviour in four contexts including work and competition. The behavioural traits were divided into three groups: working manoeuvres, working attributes and general attributes. Data from 811 respondents revealed that several behavioural traits were of high and low value to handlers and breeders across all contexts, while others were unique to only one or two contexts. For example, *cast*, *force*, *gather*, *trainable*, *confident* and *friendly* were of most value; whereas *bite*, *bark* and *back* were of less value. Further analysis revealed that respondents can be considered as coming from two main groups: firstly, handlers with a preference for specialised dogs in the utility context and, secondly, handlers focussed on the yard context, who need dogs that have a broad range of skills and that are easy to work with. This information may assist in matching handlers with suitable dogs. Future research should clarify handlers’ understanding of innate and learnt behaviours.

**Abstract:**

This study investigated the value that handlers and breeders assign to various behavioural traits in Australian livestock herding dogs. Data were obtained from 811 handlers and breeders through the ‘Australian Farm Dog Survey’. Respondents were asked to consider dogs within four contexts: utility (livestock herding in both paddocks and yards), mustering (livestock herding in paddocks and along livestock routes), yards (in and around sheds, sale-yards and transport vehicles), and trial (specifically a standard 3-sheep trial), and to rate the value of 16 working manoeuvres (movement sequences used in herding), 11 working attributes (skills or attributes used in herding) and five general attributes (personality traits ascribed to an individual dog). The most valued working manoeuvres were *cast*, *force* and *gather*. *Bite*, *bark* and *backing* were considered of little value in certain contexts, notably the trial context. Across all four contexts, the general attributes most valued in dogs were being *trainable*, *motivated*, *confident* and *friendly*, while *control* and *trainability* were the working attribute traits considered to be of most value. *Excitability* was revealed to be a ‘Goldilocks’ trait in that respondents preferred not too much or too little but a ‘just right’ amount in their preferred dog. Analysis indicated a handler preference for either specialised dogs for the utility context or dogs who are easy to work with because of a broad range of traits favoured in the yard context. These results reveal both generalities across and the need for specialisation within these four herding contexts. Further investigation may help to reveal how well handlers distinguish between innate and learnt behaviours when selecting and training livestock herding dogs. Identifying which group handlers fit into optimally may assist in selecting suitable dog–human dyads.

## 1. Introduction

The national population of working livestock herding dogs in Australia has been estimated at more than 94,000 individuals [1]. Over an average working career, the estimated economic value of these dogs’ work is over A$40,000 per individual [2]. Successful partnerships between dog and handler reflect the quality of the match between the personality and behaviour profile of the dog and the preferences, experience and skills of its handler as well as the perceived financial value of the dog [3]. 

Livestock herding dogs are routinely used to move livestock in three over-arching contexts that are also used to label specific working skill-sets: utility (both paddock and yard), mustering (paddock and livestock routes) and yard (in and around sheds, sale-yards and transport vehicles). They are selected primarily for performance and health rather than morphological traits [4], an approach that has resulted in the prevalence of a suite of behaviours thought to be stylised elements of the predatory sequence exhibited by *Canis lupus familiaris* [5,6,7]. 

Natural instinct, combined with opportunities to regularly practise and be reinforced for herding behaviours, is fundamental to any dog’s performance in a herding task. The unique triadic interaction of humans, dogs, livestock and sometimes handlers on horseback has been referred to as a ‘mutually adjusted system’ [8]. Insufficient or poor quality training may jeopardise dog and livestock welfare and compromise learning outcomes [9,10,11]. Investigations into handler–dog interactions during livestock herding training have focussed on moderating access to livestock through negative punishment (interrupting access to livestock) or positive reinforcement (allowing continued access to livestock) [5,12]. These operant techniques reveal the reinforcing value of access to livestock in dogs that have been selected to relish this sort of work [13].

Data on the ease or difficulty with which livestock herding dog handlers can condition dogs to perform certain working behaviour traits may reveal areas in which trainer education may be especially beneficial. They may also identify which traits deserve a particular focus in breeding and selection to ensure livestock herding dogs can perform the task for which they are being bred. 

Peer-reviewed studies on the behaviour of livestock herding dogs are rare (*see* [5,8,12,14,15,16]) and, among them, few are easily transferable to the Australian context. Importantly, only two studies [5,8] defined the dogs used in their studies as working dogs or from working dog lines, rather than companion dogs of herding breeds. One Australian study, which was not subject to peer-review, examined the inheritance of the behavioural trait *eye* (commonly defined as a dog’s ability to hold sheep together by staring at them) [17]. It reported that, when using a six-point scale to score *eye*, the 28 dogs tested were most likely to be scored as intermediate to or aligned with one of their parent’s scores. 

Popular literature on livestock herding in Australia suggests that certain behavioural traits such as *eye*, *force*, *boldness*, *anticipation* and *cast* are pivotal to successful herding ability [18,19]. However, the definition, interpretation, perceived value and relevance of these and other traits varies among authors [17,18,20,21,22,23,24,25]. For example, a recent study of eight Australian herding manuals identified a significant discordance in the frequency of the use of such popular terms [26]. 

The current study used a questionnaire to identify the ideal position for a livestock herding dog on the shyness–boldness continuum, the value that handlers place in the ideal dog on the expression of five general attributes; 11 working attributes; 16 working manoeuvres and the ease with which dogs can be trained to show each of these in four distinct herding contexts. A central hypothesis for the current survey study was that respondents would, having some knowledge of innate versus learnt behaviours, report innate behaviours as being more difficult to train.

## 2. Materials and Methods

The ‘Australian Farm Dog Survey’ was designed to investigate the distribution of farm dogs in Australia, their usage, their management and the views of their owners along with demographic information relating to the breeder/handler (for other publications using this survey *see* [2,3,27]). In particular, respondents were asked about five general attributes, 11 working attributes and 16 working manoeuvres in their dogs. The questions were grouped within four herding contexts: utility, mustering, yard, and competition (3-sheep trial).

Prior to publication of the survey, popular working-dog training manuals were consulted and advice was sought from members of the Working Kelpie Council of Australia to ensure that the terminology in the survey was appropriate for the target audience. A pilot distribution of the survey to 125 participants led to some minor modifications prior to widespread distribution. 

The online version of the survey was available over a three-month period from 10 March to 10 June 2013. All promotional materials relating to the survey indicated that a hard copy of the survey with a reply-paid envelope would be provided to participants if they requested one by telephone. Approval for this study was granted from the University of Sydney Human Research Ethics Committee (Approval number 15474).

The target population for the survey was livestock herding dog users across Australia. Participation was encouraged with entry into a prize draw to win commercial working-dog food at the end of the survey period. An introductory message gave participants the option to respond anonymously with an assurance of confidentiality were they to choose to leave their details to enter the prize draw.

A link to the online survey was posted on the websites of the University of Sydney [28] Meat and Livestock Australia [29] and the Working Kelpie Council of Australia (WKCA) [30]. It was advertised through stories in multiple regional newspapers, on three nation-wide television programs and in two national agricultural magazines. The committee of the 2013 Casterton Kelpie Auction (one of Australia’s leading livestock herding dog auction events) promoted the survey in a mail-out to past and current vendors and purchasers. The researchers also recruited survey participants, in person, at livestock herding dog trials during the study period.

The online version of the survey was constructed using the survey system QSmart (Torque Management Systems Limited, Auckland, New Zealand). The entire questionnaire had a maximum of 143 items assigned to 10 sections. However, participants needed to answer fewer questions if they responded in the negative to questions about certain activities, such as breeding or trialling of dogs. The logic system of the online survey permitted the redirection of participants to questions of relevance. (To view the complete survey, *see* [31].

In Early et al. [26], we defined working manoeuvres, working attributes (referred then as working skills) and general attributes: working manoeuvres represent a sequence of movements used in herding; working attributes reflect an ability used in herding; general attributes are personality traits ascribed to an individual. Where a trait might fit into both working and general attributes or might lie on the same spectrum (e.g., *boldness* and *cautiousness*), the authors made a decision into which category it should be included, based on whether testing and exploring these relationships would identify, statistically, if they are valued in different ways.

Respondents were asked to indicate the ease with which 16 working manoeuvres in the typical working dog can be trained: *cast*, *force*, *gathering*, *cover*, *backing*, *bark*, *bite*, *heading*, *hold*, *balance*, *drive*, *break*, *width*, *pull*, *lift* and *draw*. They answered using a semantic differential-type 5-point rating scale. Descriptive phrases ‘extremely easy’ and ‘almost impossible’ were used at either extreme of the scale. Respondents were advised to not provide a rating for any working manoeuvre terms that they were unfamiliar with.

The same 16 working manoeuvres were used again in the next question, which asked the respondents to indicate how valuable they considered these behaviours in livestock herding dogs. Respondents were asked to answer this question separately for up to four herding contexts in which they had experience. These included three types of work, namely, utility (generally known among trainers as all-round), mustering, and yard, and one competition context referred to as trial (i.e., working trials, generally known among trainers as arena or 3-sheep trials). Answer options included a semantic differential-type 5-point rating scale, ranging from ‘no value’ to ‘highly valuable’. Respondents unfamiliar with any working manoeuvre terms were advised to not provide a rating.

The handlers were asked to score the value of 11 working attributes—*shows eye*, *control*, *initiative* (which we described as including working independently, *keenness* and willingness), *trainable* (including tractable), *intelligent* (including sagacious, brainy and clever), *calm*, *firmness* (including strength and power), *style of work* (including width), *physical suitability* (including stamina and durability), *anticipation* and *boldness*. These attributes’ values were recorded for each working and competition environment within which the respondent had handled livestock herding dogs. Answer options included a semantic differential-type 5-point rating scale. Descriptive phrases ‘no value’ to ‘extremely valuable’ were used at either end of the scale. Respondents unfamiliar with any working attribute terms were advised to not provide a rating.

Respondents were asked to indicate the degree of expression of five general attributes they would expect to be present in the ideal dog for the working and competition environments in which they had experience. These attributes were *excitability*, *trainability*, *motivation and confidence*, *friendliness* and *cautiousness*. They were drawn, and slightly modified from, the “Big Five” personality traits identified by Ley et al. [32]. Respondents answered using a semantic differential-type 5-point rating scale ranging from ‘none’ to ‘a very high degree’ at each end point with ‘a moderate degree’ at the midway point. Respondents unfamiliar with any general attribute terms were advised to not provide a rating.

The final question asked handlers to indicate, on a 100-point visual analogue scale, the balance of shyness–boldness expression they would expect the ideal dog to exhibit for each herding context in which they had experience, where zero was maximum shyness and 100 was maximum boldness. Specifically, they were asked: *Please indicate, by moving the sliding scale/marking on the scale, the balance of shyness and boldness that the ideal dog would exhibit*. As a guide, the descriptive phrases ‘extremely shy’ and ‘extremely bold’ were used at either extreme of the scale. Respondents unfamiliar with the concept of shyness–boldness expression were advised to not provide a rating.

Genstat Version 16 (VSN International, Hemel Hempstead, UK) was used for statistical analysis of shyness–boldness expression results. REML analysis was performed using the variate means of shyness–boldness expression for each herding context: utility, mustering, yard and trial. This permitted assessment of whether differences in ideal shyness–boldness expression across the four contexts were significant.

Individual handler optima on the shyness–boldness expression in the ideal dog for each of the four herding contexts were gathered and descriptive statistics collated. The same approach was taken to the amount of five general attributes in the ideal dog; the 11 working attributes in their dogs; the 16 working manoeuvres and the training ease of each working manoeuvre. 

To explore the influence of context on handler preferences, a hierarchical cluster analysis based on Gower distance (as all variables in this study were ordinal; this corresponded to Manhattan distance) was conducted using the hclust function of the R statistical and computing software [33].

A Pearson’s chi-squared test was performed to assess the significance of the results.

## 3. Results

### 3.1. Respondent Demography

Of the 812 livestock herding dog handlers and breeders who completed the survey, 563 were male and 249 were female. Most respondents were aged 50–59 years (*n* = 213), followed by 60–70 years (*n* = 182), 40–49 years (*n* = 165), 30–39 years (*n* = 120), 20–29 years (*n* = 120), and over 70 years (*n* = 44). There were more respondents who did not breed dogs (non-breeders, *n* = 451) than who did (breeders, *n* = 361). More than half of respondents had acquired knowledge of dog training beyond ‘on-the-job’ experience (experience only, *n* = 302; further education, *n* = 510), but only 19 of these respondents had completed a certified course. The remainder reported having attended dog-training schools and/or read dog-training books.

### 3.2. Herding Context Experience

Among the 811 livestock herding dog handlers and breeders who selected having experience in one or more of the four herding contexts, the following contexts (and combinations of contexts) were selected: mustering/yard/utility (*n* = 241), mustering/yard (*n* = 169), utility (*n* = 125), mustering/yard/trial/utility (*n* = 96), mustering (*n* = 85), mustering/utility (*n* = 33), mustering/yard/trial (*n* = 14), yard (*n* = 13), mustering/trial (*n* = 10), mustering/trial/utility (*n* = 10), trial (*n* = 5), yard/utility (*n* = 4), trial/utility (*n* = 3), yard/trial (*n* = 2), and yard/trial/utility (*n* = 1). Totals for each herding context were: mustering (*n* = 658), yard (*n* = 540), utility (*n* = 513) and trial (*n* = 141).

### 3.3. Preferred Balance of Shyness–Boldness Expression in the Ideal Dog

**Figure 1:**
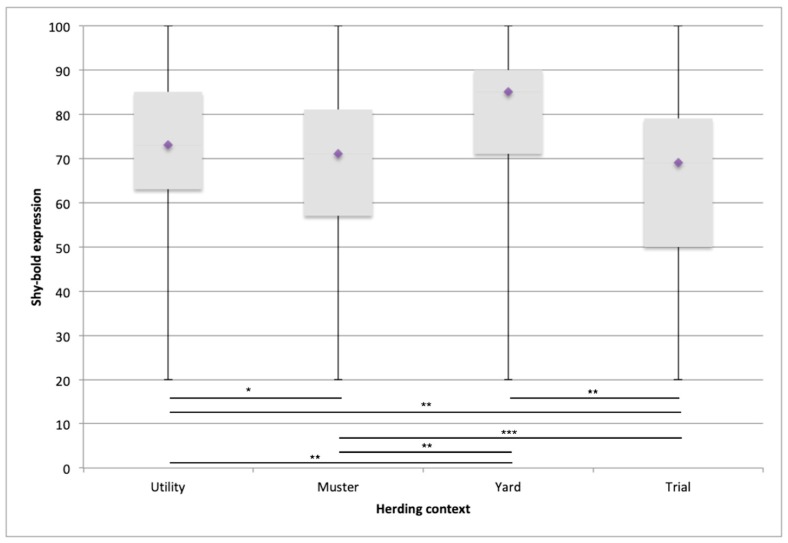
Boxplot of shyness–boldness expression in the ideal dog per herding context: the box spans the interquartile range of the values; middle 50% of the values lie within the box with a diamond indicating the mean. The whiskers extend beyond the box to represent the range of the data. Respondents marked shyness–boldness expression on a visual scale that used the descriptive phrases ‘extremely shy’ and ‘extremely bold’ at either end. * *p* = 0.006 ** *p* < 0.001 *** *p* = 0.004.

The highest boldness expression in the ideal dog selected by respondents was in the yard context, followed by utility, mustering and trial (means of 79.12, 72.11, 69.77, and 65.43, respectively). Between contexts, differences in mean value were statistically significant between trial and mustering (*p* = 0.004), mustering and utility (*p* = 0.006), highly significant between utility and trial (*p* < 0.001) and yard and all other contexts (mustering/trial/utility, *p* < 0.001) (see Figure 1).

### 3.4. Amount in the Ideal Dog

#### 3.4.1. Amount of General Attributes in the Ideal Dog for Utility Work

**Figure 2:**
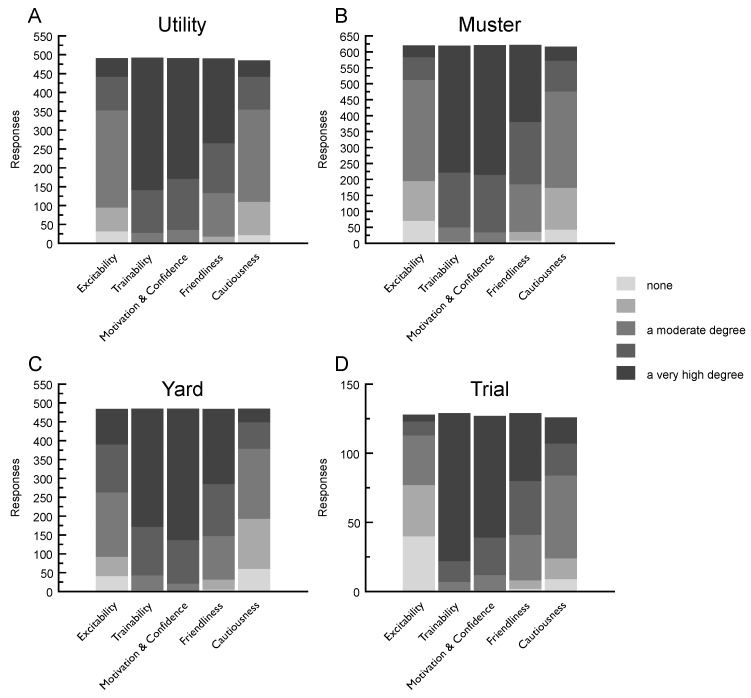
Amount of five (5) general attributes in the ideal dog across the four herding contexts. Respondents’ ratings: **A**—utility; **B**—mustering; **C**—yard; **D**—trial.

For the utility context, most respondents selected ‘a very high degree’ for the general attributes of being *trainable* (*n* = 351; out of 492) and *motivation and confidence* (*n* = 320; out of 491). Meanwhile, over half the respondents selected ‘a moderate degree’ (midway on the five-point scale) for *excitability* (*n* = 258; out of 491) and *cautiousness* (*n* = 245; out of 485) (see Figure 2).

#### 3.4.2. Amount of General Attributes in the Ideal Dog for Mustering Work

For the mustering context, similar to the utility context, most respondents selected ‘a very high degree’ for *trainable* (*n* = 397; out of 619) and *motivation and confidence* (*n* = 406; out of 621). In comparison, for *excitability* (*n* = 317; out of 620) and *cautiousness* (*n* = 302; out of 616), no respondent selected more than ‘a moderate degree’ in the ideal dog (see Figure 2).

#### 3.4.3. Amount of General Attributes in the Ideal Dog for Yard Work 

Most respondents who supplied data for the yard context, similar to both utility and mustering contexts, selected ‘a very high degree’ for *trainable* (*n* = 313; out of 485) and *motivation and confidence* (*n* = 348; out of 485). Unlike the utility and mustering context results, respondents selected increased amounts of *excitability* and similar *cautiousness* in the ideal dog (see Figure 2).

#### 3.4.4. Amount of General Attributes in the Ideal Dog for Herding Trials

In the trial context, 107 out of 129 respondents selected ‘a very high degree’ for *trainability*. Additionally, more than half of respondents selected ‘a very high degree’ for *motivation and confidence* (*n* = 88; out of 127). *Excitability* was considered least useful in the trial context with ‘none’ selected relatively more by respondents (*n* = 40; out of 128) than the working contexts: mustering (*n* = 70; out of 620), yard (*n* = 41; out of 484) and utility (*n* = 32; out of 491) (see Figure 2).

### 3.5. Value of Working Attributes

#### 3.5.1. Value of Working Attributes—Utility

**Figure 3:**
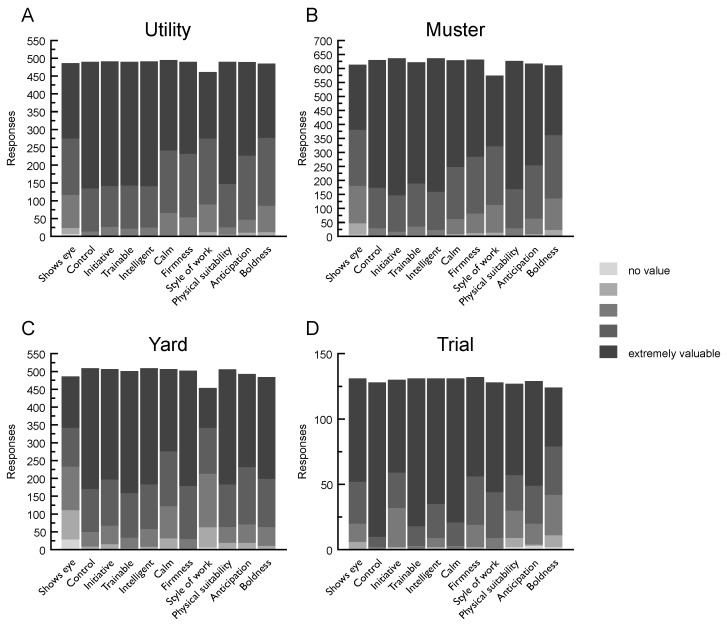
The value of eleven (11) working attributes across the four herding contexts. Respondents’ ratings: **A**—utility; **B**—mustering; **C**—yard; **D**—trial.

Respondents scored five working attributes as ‘extremely valuable’: *control* (*n* = 355; out of 490), *intelligent* (*n* = 350; out of 491), *initiative* (*n* = 349; out of 491), *trainable* (*n* = 347; out of 490), and *physical suitability* (*n* = 343; out of 490). *Style of work* received the fewest ratings of ‘extremely valuable’ overall (*n* = 186; out of 461). Most respondents selected one of the two highest ratings for *boldness* (‘extremely valuable’ *n* = 208, followed by the next point on the scale (unlabeled) *n* = 191; out of 485) (see Figure 3).

#### 3.5.2. Value of Working Attributes—Mustering

*Initiative* and *Intelligence* were considered ‘extremely valuable’ by 489 and 476 (both out of 636) respondents. Of the 611 respondents who assigned scores to this context for *boldness*, 475 selected the two highest ratings (see Figure 3). 

#### 3.5.3. Value of Working Attributes—Yard

Respondents considered the most valuable working attributes in the yard context *trainable* (‘extremely valuable’ *n* = 342; out of 501), *control* (‘extremely valuable’ *n* = 339; out of 509), *intelligence* (‘extremely valuable’ *n* = 326; out of 509), *firmness* (‘extremely valuable’ *n* = 323; out of 502) and *physical suitability* (‘extremely valuable’ *n* = 323; out of 506). They considered *eye* and *style of work* as being of least value, with responses more evenly spread across the ordinal scale compared to all other working attributes (see Figure 3).

#### 3.5.4. Value of Working Attributes—Trial

*Control* (*n* = 118; out of 130), *trainable* (*n* = 113; out of 131) and *calm* (*n* = 110; out of 131) were scored as ‘extremely valuable’ by most respondents when describing the ideal trial dog. *Boldness* was rated the least valuable of the working attributes assessed (see Figure 3).

### 3.6. Value of Working Manoeuvres

#### 3.6.1. Value of Working Manoeuvres—Utility

**Figure 4:**
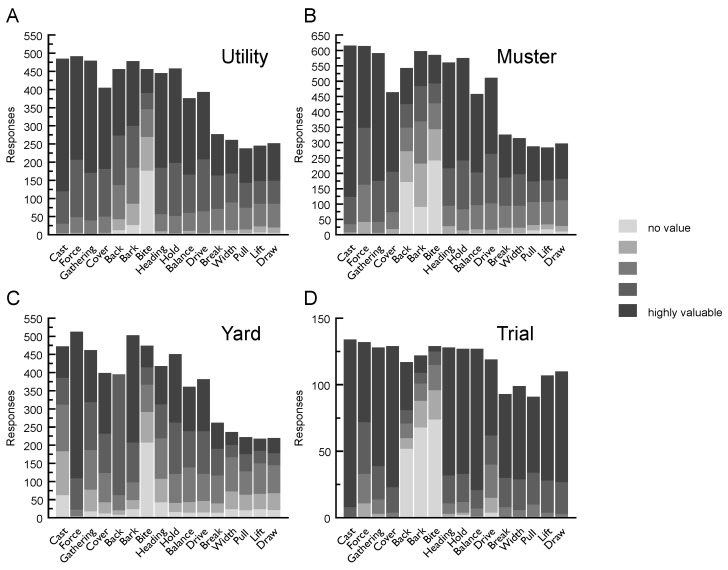
The value of sixteen (16) working manoeuvres across the four herding contexts: Respondent’s ratings: **A**—utility; **B**—mustering; **C**—yard; **D**—trial.

Respondents describing the ideal utility dog considered *cast* to be of highest value (‘highly valuable’ *n* = 365; out of 485 responses), and *bite* received the highest score for ‘no value’ (‘no value’ *n* = 177; out of 456). *Break*, *width*, *pull*, *lift* and *draw* received fewer than 300 responses, likely indicating that knowledge of these terms was limited to a sub-set of survey respondents (see Figure 4).

#### 3.6.2. Value of Working Manoeuvres—Mustering

Six working manoeuvres were considered ‘highly valuable’ by most respondents in the mustering context: *Cast* (*n* = 492; out of 616), *gathering* (*n* = 415; out of 591), *cover* (*n* = 259; out of 463), *heading* (*n* = 344; out of 561), *hold* (*n* = 333; out of 575) and *balance* (*n* = 255; out of 458). *Bite* was considered to be of ‘no value’ (*n* = 242) or of limited value (*n* = 102) by most respondents (total *n* = 584). Responses on the value of *backing* were spread across the five-point scale (‘extremely valuable’ *n* = 117, *n* = 77, *n* = 77, *n* = 100, ‘no value’ *n* = 172; out of 543) (see Figure 4).

#### 3.6.3. Value of Working Manoeuvres—Yard

In the yard context, *force* and *bark* were considered ‘highly valuable’ (*n* = 404; out of 513, *n* = 295; out of 503) by most respondents. No one considered *back* ‘highly valuable’ (*n* = 0; out of 395). *Bite* was scored as having ‘no value’ by nearly half of respondents (*n* = 208; out of 474). Similar to the mustering context results, *break*, *width*, *pull*, *lift* and *draw* each received fewer than 262 responses, compared to more than 361 each for the other traits (see Figure 4).

#### 3.6.4. Value of Working Manoeuvres—Trial

The highest value traits in the trial context were *cast* (‘highly valuable’ *n* = 126; out of 134), *balance* (‘highly valuable’ *n* = 106; out of 127), *cover* (‘highly valuable’ *n* = 106; out of 128), *heading* (‘highly valuable’ *n* = 96; out of 128) and *hold* (‘highly valuable’ *n* = 94; out of 127). Most respondents scored *bite* (‘no value’ *n* = 74; out of 129) and *bark* (‘no value’ *n* = 68; out of 122) as ‘no value’ (see Figure 4).

### 3.7. Trainability of Working Manoeuvres

**Figure 5:**
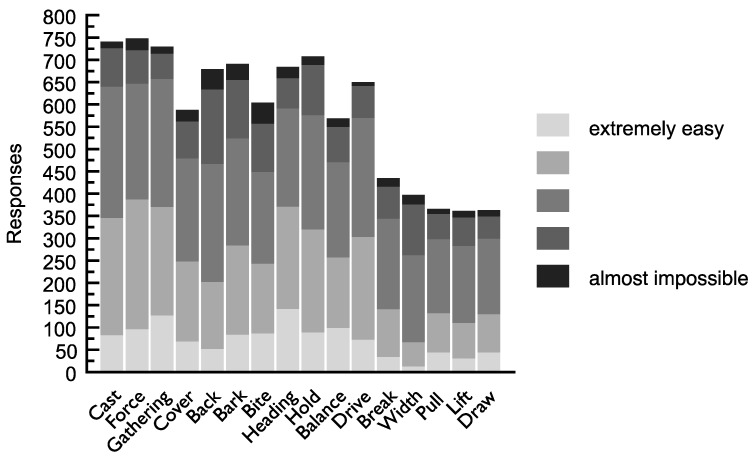
Reported ease of training for sixteen (16) working manoeuvres.

Among the 16 working manoeuvres, all but two traits were scored by respondents at the midway point between ‘extremely easy’ and ‘almost impossible’. These were *force* (first two points at the end of the scale including ‘extremely easy’ *n* = 387; out of 748) and *heading* (first two points at the end of the scale including ‘extremely easy’ *n* = 371; out of 684), indicating respondents’ possible awareness of these traits being innate behaviours. *Break* (*n* = 435), *width* (*n* = 397), *pull* (*n* = 366), *lift* (*n* = 361) and *draw* (*n* = 363) received fewer than 435 responses. For all the other traits measured, there were at least 569 responses each: *Cast* (*n* = 741), *force* (*n* = 748), *gather* (*n* = 730), *cover* (*n* = 588), *back* (*n* = 679), *bark* (*n* = 691), *bite* (*n* = 604), *heading* (*n* = 684), *hold* (*n* = 708), *balance* (*n* = 569), *drive* (*n* = 650) (see Figure 5).

### 3.8. Cluster Analysis

**Figure 6:**
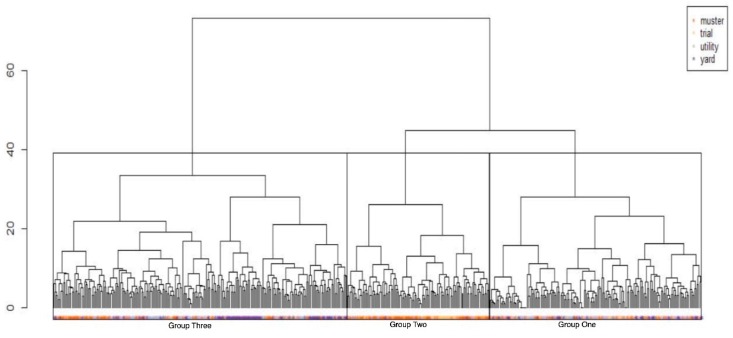
Cluster dendogram showing hierarchical, agglomerative clustering of Euclidean Distances of preference scores for analysed traits. The type of work or competition for which a trait was favoured by respondents is indicated by: Green = utility, Red = mustering, Blue = yard, Yellow = trial. Dendogram shows, from left to right, Group Three, Group Two, Group One.

The hierarchical cluster analysis resulted in three groups: Group Three (most common preference pattern), Group Two (the smallest preference pattern) and Group One (intermediate to Groups Two and Three) (see Figure 6).

**Figure 7:**
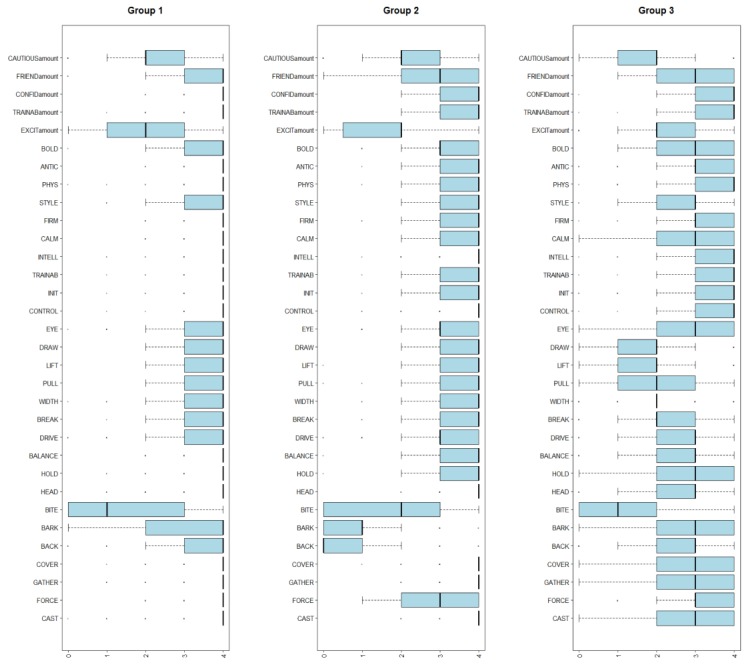
Cluster group preferences by trait: Boxplot demonstrating the different medians, interquartile ranges and whisker lengths of preferences for each trait in the three clusters of respondents shown in Figure 6.

Group One owners prioritised cast, force, gathering, cover, heading, hold, balance, firmness, calmness, intelligence, trainability, initiative, control, anticipation, physical suitability and confidence (see Figure 7). 

Group Two owners prioritised *cast*, *gathering*, *cover*, *heading*, *control* and *intelligence*. *Back*, *bark* and *excitability* were clearly not preferred in this group (see Figure 7). 

Group Three owners prioritised a more balanced approach to the value and amount of each trait. They were less concerned with *draw*, *lift*, *pull*, *width* and *break* (see Figure 7). 

Across all three groups, *bite* was consistently less in demand than the other traits analysed.

**Figure 8:**
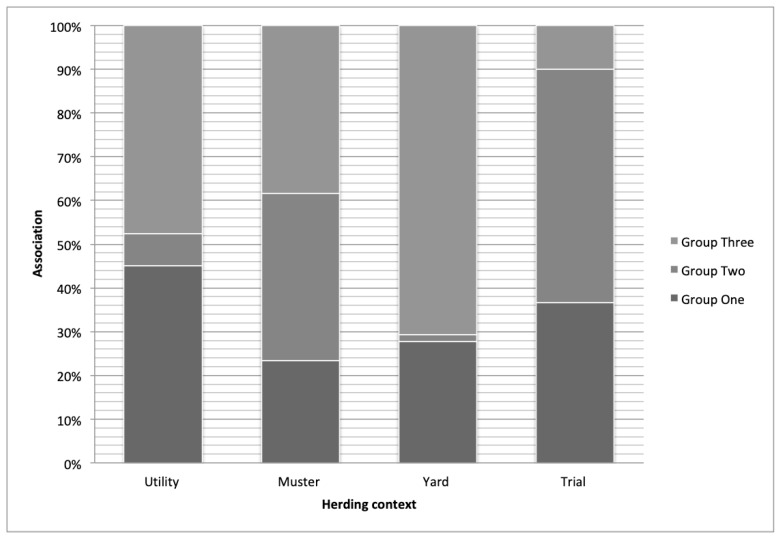
Associations of each cluster group with working and competition contexts. Each group would be expected to have equal representation across each herding context; the chart indicates which group’s preferences were over or underrepresented for each herding context.

When the three groups were analysed across the four herding contexts (see Figure 8), using Pearson’s chi-square test (chi-squared = 136.21, df = 6), highly significant (*p* < 0.01) preferences were apparent for particular contexts. Group One’s trait preferences were overrepresented for utility and underrepresented for mustering. Group Two’s trait preferences were overrepresented for mustering and trial, while underrepresented for yard and utility. Group Three’s preferences were overrepresented for yard and underrepresented for mustering and trial.

## 4. Discussion

Although surveys of owners and/or experts have been previously used to develop behavioural profiles of companion dog breeds [34,35], to our knowledge, this is the first to assess the relative value of personality and working traits in livestock herding dogs. 

By identifying the most valuable working and personality traits across multiple herding contexts, the current results help to show how these traits influence successful movement of livestock while also identifying traits that could enhance both context-specific and general breeding programs. Genetic analysis from the hunting dog sector in Sweden has shown that, for at least six traits, if Best Linear Unbiased Prediction breeding values were used instead of phenotype, genetic gain would be 89% higher [36]. With the sequencing of the canine genome [37], molecular genetics provides the opportunity to identify suitable livestock herding dogs at an earlier age than behavioural assessment currently offers.

Of the four herding contexts surveyed, mustering was selected as the context in which most respondents had experience with handling livestock herding dogs. This was followed by yard and utility, with only a small number of respondents involved in the trial (competition) environment. These distributions reflect prevalent Australian working conditions in that livestock are often collected over large areas of farmland prior to being managed within yards. Whether this Australian distribution of potential respondents and the current resultant data are similar in other large-scale livestock-producing countries requires further investigation.

### 4.1. Shy–Bold

The ideal reported balance of shyness–boldness expression significantly differed across the four herding contexts. Livestock herding dogs working in the yard context were considered by respondents to require a higher level of *boldness* than in the utility and mustering contexts, followed by the trial context. However, the middle 50% of responses overlapped between each context. This finding reflects the necessary blend of confidence, motivation, composure and resilience that presumably make up the term boldness, the so-called super trait that yard dogs require to be successful in this close-up, threatening, high risk environment. The expression of *boldness* appears to be a simple way for respondents to differentiate between the type of dog required between each herding context.

### 4.2. General Attributes

For general attributes, handlers and breeders across all herding contexts reported that the ideal dog has a high degree of *trainability*, *motivation and confidence* and *friendliness*. The cluster analysis also identified similarities across the three groups for the amount preferred of these traits. The results for *excitability* highlighted one of the more interesting findings for the general attributes group. Across all contexts and the three cluster analysis groups, respondents identified this as the ‘Goldilocks’ trait—not too little, not too much, but ‘just right’; the term Goldilocks principle or effect is referred to in other research fields including economics and education to describe contexts that seek balance [38,39]. Finding a balance for *excitability* is not unique to livestock herding dogs, for example it is also noted in guide dogs [40]. However, improvements in selective breeding for this trait may assist future research about behavioural tendencies being undertaken among service dogs [41,42]. 

Respondents’ preference for a dog that is easy to work with, through long hours, and often as the handler’s only companion in conducting their work has implications for the welfare of these dogs. Specifically, livestock herding dogs that do not meet handlers’ expectations of being ‘trainable, motivated, confident and friendly’ could be at more risk of becoming so-called behavioural wastage (being discarded from the industry because of poor performance related to behaviour, rather than physical inadequacy) [43]. ‘A moderate degree’ of *excitability* and *cautiousness* was preferred, reflecting respondents’ awareness of the potential for high levels of these traits to compromise the successful working performance of a dog. This preference for trait-specific expressions of certain qualities is a complex finding. A common difficulty in breeding or selection of livestock herding dogs is consistency between what one handler or breeder and another considers to be ‘the right amount’ of a trait expressed in a dog. 

### 4.3. Working Attributes

As far as working attributes are concerned, the current study revealed both similarities and differences between the four herding contexts and confirmed, by the cluster analysis, the key working characteristics preferred and valued by handlers. Across all contexts, the high values assigned to *control* and *trainable* indicate the importance of these traits in allowing handlers and breeders to breed, rear and train the best dogs for each herding context. While dogs working at a distance from their handler are not unique to livestock herding, what is specialised to livestock herding dogs is the concurrent gathering and movement of livestock to the handler. The understanding of respondents that dogs in this context pose a risk to themselves (e.g., placing themselves in dangerous positions leading to injury) and the livestock (e.g., injuring livestock due to poor herding technique) should not be underestimated. For both mustering and utility contexts, respondents reported that dogs with *initiative*, *intelligence* and *physical suitability* were of most value to them. These traits reflect the complex and demanding nature of work in these contexts and the requirement for handlers to both direct their dogs, when needed, and rely on them to perform independently, as required. In the yard context, most respondents also assigned high value to *firmness* and *physical suitability*. *Boldness*, a personality trait often referred to in the peer-reviewed canine behaviour literature (e.g., [44,45]), was considered of less value than the other working attributes in the utility, mustering and trial contexts, yet was one of the highest value attributes in the yard context. These results reflected those obtained from the shyness–boldness expression question. Similarly, *shows eye*, a core attribute of herding dogs that has clear analogues in the predatory sequence [8], was considered of high value only in the trial context. These apparent anomalies suggest that the demands of herding work peak within the broad yard context, which often requires dogs to move fearful livestock at a close distance, through tight spaces, repeatedly. 

### 4.4. Working Manoeuvres

The value of the 16 working manoeuvres across the four herding contexts revealed some key similarities between the four herding contexts. In the utility, mustering and trial contexts, *cast*, *force*, *gathering*, *cover*, *heading*, *hold*, *balance* and *drive* were consistently of high value to respondents whereas *force* and *bark* were of extreme value to respondents compared to the other manoeuvres. While these results are not surprising, they represent a selection of manoeuvres on which breeders or handlers may focus to ensure their dogs meet expectations when working. Notable exceptions were *bite* and *backing*. Across all four contexts, many respondents assigned ‘no value’ for *bite* while *backing* was assigned ‘no value’ by many respondents in the mustering and trial contexts, reflecting their relative unhelpfulness in these contexts. Additionally, throughout the cluster analysis, *bite*, being the trait of least value, revealed awareness among respondents that the ideal dog should show limited expression of this trait.

The current study clearly identified manoeuvres whose reported value differed from one context to the next. For example, *cast* was rated as the most valuable in utility, mustering and trial contexts, but as only of general value in the yard context. Many respondents consistently rated *bite* as having ‘no value’ across each context. 

The cluster analysis provided further insight into respondents’ thoughts on working manoeuvres. Among handlers, there appear to be two main groups who require all-rounder type dogs. The first group (Group One) were those who have a clear preference for a select group of traits they need to perform the work with a focus on the utility context. This group also appears to have a keen interest in the skills their dogs need to be successful. The second group (Group Three) were those handlers who want ‘jack of all trades’ dogs that possess a broad range of working manoeuvres and consider most manoeuvres to have only moderate value. This group reported primarily on the yard context. Further studies are required to differentiate between the first group, who may be experienced students of livestock herding dogs with well-developed ideas of the most valuable traits to have a successful dog, and the second group, who seem to represent the majority of yard-based Australian livestock herding dog handlers who are primarily outcomes-focused. These results have provided an additional layer to this survey’s findings: that, while there appears to be a move towards a need for specialist livestock working dogs on the whole, generalities remain and there is still a need for those who primarily work in the yard context to have dogs with a broad, albeit average, skill set.

### 4.5. Ease of Training Working Manoeuvres

When respondents were asked how easy or difficult it is to train a group of working manoeuvres, the hypothesis was that respondents of the survey would select innate behaviours as being more difficult to train. The results indicate that, overall, the manoeuvres were neither easy nor difficult to train. This may reflect our use of scales that had insufficient granularity to detect real differences between ease of training. Two manoeuvres that appear to be elements of the predatory sequence, *force* and *hold*, were reported by respondents to be easier to train than the other manoeuvres. It is possible that some handlers with a good understanding of livestock herding dog behaviour may find innate behaviours easier to train or shape, while others may credit their own training skills for any apparent ease of training certain innate behaviours. For accomplished trainers, creating scenarios for a livestock herding dog to practice and trigger these innate behaviours may make them relatively easy to train by simply fine-tuning a natural behaviour. In Australia, mean failure rates of herding dogs in training have been estimated to be at least 20% [2]. Improving understanding of the practical application of behavioural science findings as they pertain to livestock herding dogs may boost training outcomes [46]. This approach has met with considerable success in horse training through the nascent discipline of equitation science [47]. Additionally, identifying consistently successful trainers with low failure rates, who can provide assistance to fellow trainers through sharing their knowledge, may also have merit.

While the current results provide some helpful information on the relative value of working and personality traits in the four herding contexts, they do contain some limitations. Among all three non-competition contexts, the manoeuvres *break*, *width*, *pull*, *lift* and *draw* were unfamiliar to many respondents in the working context. This is most probably because these terms are often used in competition and technical literature but rarely in the practical, working contexts. An additional limitation is that the current survey did not explore the specific reasons for why individual manoeuvres were either of high or low value to each respondent or why some respondents did not assign a rating or score to a given quality. While the terms presented in the current survey were identified as those most commonly used in relevant manuals [26], there is scope to further investigate why these working manoeuvres provide value within each herding context.

Furthermore, due to the large number of questions, it was apparent that respondents missed answering parts of questions, developed survey fatigue or were not familiar with some of the terms or traits presented. Improved survey design, including attempts to verify whether respondents deliberately failed to answer part of any given question, may assist in reducing missing data. A further limitation that we acknowledge as a potential source of lost detail among the data arose in the general attributes questions where, for consistency with Ley et al. [32], the personality dimensions *motivation* and *confidence* were grouped together. Because of this, it was not possible to determine whether respondents were selecting just one or both terms in their response. As such, this result should be interpreted with some caution. The question on training ease of the 16 working manoeuvres attempted to tease out respondents’ understanding of the difference between innate and learnt behaviours in relation to livestock herding. While the results indicate some variability in understanding by respondents, a published analysis of a different section of results from the same survey, focusing on dogmanship, found a general lack of understanding of learning theory and training principles among the current respondents [27]. Further investigation into survey respondents’ understanding of the behavioural underpinnings of these working manoeuvres may assist interpretation of this section of the survey.

There is limited research analysing the effect of sex on behavioural traits in livestock working dogs. While data on sex in relation to the behavioural traits in this study was not collected, *confidence* and *boldness* are examples where sex differences could be explored in future studies. Additionally, sex differences in training ease among livestock working dogs may provide interesting comparisons to other working dog sectors [48].

## 5. Conclusions

This survey identified preferred levels of boldness and those general attributes, working attributes and working manoeuvres of greatest value to most Australian handlers and breeders across four herding contexts. These results highlight similarities in the attributes and manoeuvres valued across these contexts but also the need for dogs working in an individual context to develop specialised skills. However, there was, among the respondents, a sub-group of handlers with a focus on the yard context, who need dogs with a broad range of skills that are easy to work with. The most valued general attributes were *trainable*, *friendly*, *motivation* and *confidence*. The most valued working attributes were *control* and *trainable* while the most valued working manoeuvres included *cast*, *force*, *cover* and *gathering*. Further investigation is required to explore handlers’ understanding of the distinction between innate and learnt behaviours in training livestock herding dogs.
